# Use of the Recombinant Tissue Plasminogen Activator in the Management of Complex Infected Intraperitoneal Fluid Collection

**DOI:** 10.1155/2019/8943837

**Published:** 2019-05-26

**Authors:** Aibek E. Mirrakhimov, Michel Boivin

**Affiliations:** University of New Mexico School of Medicine, Division of Pulmonary, Critical Care and Sleep Medicine, Albuquerque, NM, USA

## Abstract

Intra-abdominal infections and infected fluid collections are the result of local infection typically involving the gastrointestinal or genitourinary tract. These infections are usually polymicrobial. The management of such patients should include source control and appropriate antimicrobial therapy. Source control is essential and can be achieved either surgically or by percutaneous drainage of intra-abdominal fluid collection. Interventional radiology drainage of intra-abdominal fluid collections may be especially important in patients with high surgical risk or in patients who refuse surgery. Below, we present a case of successful use of recombinant intraperitoneal tissue plasminogen activator use in a patient with a complex polymicrobial fluid collection.

## 1. Introduction

Intra-abdominal infections and infected fluid collections are the result of infection typically involving the gastrointestinal or genitourinary tract [1]. These infections are usually polymicrobial. The management of such patients should include source control and targeted antimicrobial therapy. Source control is essential and can be achieved either surgically or by percutaneous drainage of intra-abdominal fluid collection. Interventional radiology drainage of intra-abdominal fluid collections may be especially important in patients with high surgical risk or in patients who refuse surgery. Below, we present a case of successful intraperitoneal use of recombinant tissue plasminogen activator (rtPA) use in a patient with complex polymicrobial fluid collection.

## 2. Case Presentation

A 54-year-old male with a history of influenza infection complicated by severe acute respiratory distress syndrome (ARDS) requiring veno-venous extracorporeal membrane oxygenation (ECMO) support for 64 days was discharged to a long-term acute care facility from our hospital. Unfortunately, seventeen days after discharge, the patient deteriorated and was readmitted with complaints of abdominal pain and nonbloody vomiting. The patient's condition rapidly progressed to septic shock requiring vasopressor support. Right upper quadrant ultrasound, computed tomography (CT) scan, and magnetic resonance cholangiography were negative for cholangitis, cholecystitis, or other acute intra-abdominal surgical pathologies. Because of worsening hemodynamic status, the patient was taken to the operating room. Initially, diagnostic laparoscopy was performed, but due to difficulties with the insufflation, it was converted into laparotomy. The gallbladder was found to be necrotic and perforated. The patient underwent subtotal cholecystectomy because of the inability to remove the gallbladder infundibulum because of its strong adherence to the duodenum. The pathology report came back as chronic cholecystitis.

**Figure 1:**
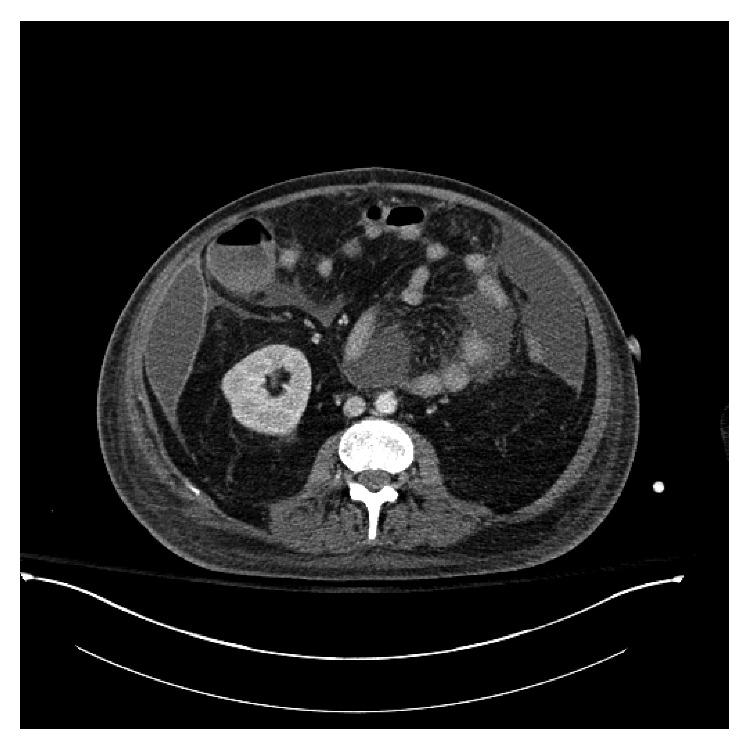
CT scan showing complex appearing right-sided intra-abdominal fluid collection.

**Figure 2:**
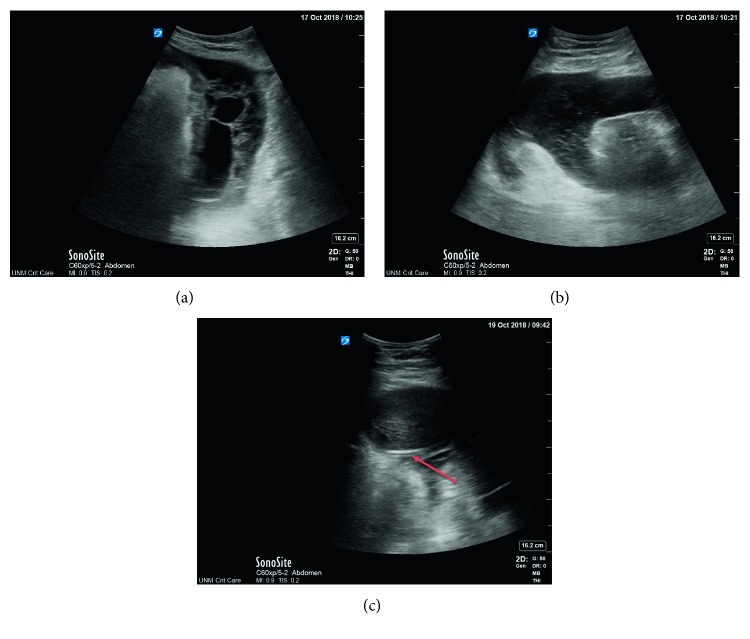
(a) Portable abdominal ultrasound showing complex intra-abdominal fluid collections with septations and hyperechoic material. (b) Portable abdominal ultrasound showing complex intra-abdominal fluid collections with septations and hyperechoic material. (c) Portable ultrasound showing pigtail catheter (arrow) inside the intra-abdominal fluid collection.

Approximately three weeks after surgery, the patient started to have worsening abdominal pain, intolerance to enteral nutrition, and recurrent signs of sepsis. The patient was started on systemic antibiotics and antifungal therapy. Repeat CT scan of the abdomen and pelvis with intravenous contrast showed extensive peritoneal thickening and enhancement in the right perihepatic region and simple appearing left-sided ascitic fluid (see Figure 1). Paracentesis of the left-sided fluid collection demonstrated an elevated WBC but no organisms. The patient underwent an imaging-guided percutaneous pigtail catheter placement into the perihepatic fluid collection. The fluid culture was positive for *Enterococcus faecalis*, *Candida tropicalis*, and *Klebsiella oxytoca*. Infectious disease consultation was obtained. The pigtail drain output was minimal (approximately 10–15 milliliters for every 24 hours), and the patient's tachycardia and marked leukocytosis persisted. Bedside ultrasound was performed which showed proper pigtail drain location within the fluid (see Figure 2(a)) and complex appearing intra-abdominal fluid with septation (see Figures 2(b) and 2(c)). After consultation with surgical team, the decision was made to instill rtPA via the pigtail catheter into the infected fluid. The patient received 4 mg of rtPA once a day for three days in total. The output over several days increased, and approximately 1 liter of fluid was drained. The patient tolerated the intra-abdominal rtPA well with no hemodynamic, hematological, or short-term intra-abdominal complications. Subsequently, the pigtail catheter was removed due to the resolution of the fluid collection. The patient made a gradual but full recovery and did not require subsequent laparotomy or drainage.

## 3. Discussion

rtPA is now conventionally used in the management of pleural empyema in order to improve fluid drainage and avoid decortication [2]. The mechanism of action of thrombolytics is believed to be mediated by decreasing the fluid viscosity and resultantly improved drainage [3]. However, the literature on the use of locally instilled rtPA among patients with complex infected intra-abdominal fluid collections is relatively scant.

Lahorra et al. studied the safety and efficacy of intracavitary urokinase among 26 patients with 31 abscesses [4]. There were 21 retroperitoneal and eight intraperitoneal abscesses in this patient population. The authors reported a high success rates for intracavitary urokinase (86% and 88% for retroperitoneal and intraperitoneal fluid collections, respectively) and no significant adverse events. In a later study, the same group studied the role of intracavitary urokinase in abscess drainage in a randomized trial [5]. The authors demonstrated that the urokinase group had decreased length of stay and costs compared to saline.

Beland et al. performed a retrospective analysis of 43 patients with 46 intra-abdominal abscesses managed with percutaneous catheters [6]. rtPA dose was 4–6 mg administered twice a day for three days. Most patients (89.1%) achieved complete evacuation of abscess, whereas 10.9% required surgery. Unfortunately, 6.5% of the abscesses recurred after catheter removal. It is important to note that no patients developed bleeding complications during the intraperitoneal rtPA administration.

Laborda et al. randomized 100 patients referred for image-guided abscess drainage to urokinase and saline [7]. The success rates were similar between the saline and urokinase groups, but patients managed with urokinase had lower costs, days of drainage, and hospital stay. Of note, intracavitary urokinase was tolerated well with no adverse effects reported.

Falsarella et al. retrospectively studied 51 patients requiring percutaneous catheter placements for abdominopelvic abscesses [8]. The dosage range of rtPA was 2–10 mg, mean treatment duration was 2.6 days, and mean time from t-PA instillation to catheter removal was 7.7 days.

Similarly, to the above reports, our patient tolerated the intraperitoneal rtPA injection well with no adverse reactions. Of note, the fluid drainage increased significantly after the rtPA was administered. This case illustrates that intracavitary thrombolytics are an important alternative therapy for infected intra-abdominal fluid collections to improve fluid drainage. They should be considered in the management of patients who are poor candidates for surgical therapy of their collections.
